# Supplementary material for: Differential Effects of Patient Navigation across Latent Profiles of Barriers to Care among People Living with HIV and Comorbid Conditions
Source: J Clin Med. 2022 Dec 23;12(1):114. doi: 10.3390/jcm12010114 (PMC9820894; doi:10.3390/jcm12010114)
Supplement: Supplementary file 1 [file jcm-12-00114-s001.zip › jcm-2039348-supplementary.pdf]

Supplementary Materials

LPA Group Differences

Differences in latent profiles by gender, race, and southern/non-southern residence were assessed using a multiple group LPA with the KNOWNCLASS option in Mplus. Each indicator was tested by comparing a model that constrained the indicator to be equal across groups with a model that freed the indicator across groups. Differences were tested using a likelihood ratio test with a significance level of α=0.05. Group differences were incorporated into the model by adding paths from the group to the specific indicators exhibiting variation. For example, if females were found to have lower income than males, a path from gender to the income indicator was added to the model.

The 3-profile LPA solution showed slight differences by race, gender, and southern/non-southern residence. Similar profiles were displayed by gender overall (Figure 2), but there were differences in several indicators. Males showed a higher average score for discrimination compared to females, particularly for the HB-DAV profile (std mean: 5.78 vs 4.85). They also had a higher probability of housing instability than females across all profiles (29.7% vs 17.4% for those with LB, 47.1% vs 40.7% for those with HB-AV, and 60.7% vs 45.7% for those with HB-DAV). For other indicators, females had higher levels than males. The most marked difference was observed in having a history of abuse across all latent profiles compared to men (46.9% vs 31.6% for those with LB, 84.9% vs 54.9% for those with HB-AV, and 90.6% vs 66.6% for those with HB-DAV). Similarly, women were more likely to have low income (90.1% vs 69.0% for those with LB, 90.0% vs 74.1% for those with HB-AV, and 88.4% vs 76.5% for those with HB-DAV) and lack transportation (92.3% vs 82.3% for those with LB, 98.8% vs 91.7% for those with HB-AV, and 100% vs 92.1% for those with HB-DAV). A history of intimate partner violence was more likely among women than men, but only for the HB-AV (80.0% vs 54.7%) and HB-DAV (83.6% vs 75.4%) profiles. Females had greater scores of social support for the LB (std mean: 2.67 vs 2.53) and HB-AV (std mean: 2.37 vs 2.01) profiles. All of the gender differences described were uniform, meaning that they were in the same direction across latent profiles.

Profile differences by race (Figure 3) were observed for 11 barriers to care: food insecurity, lack of transportation, medical mistrust, discrimination, low access to care, lack of social support, history of intimate partner violence, unemployment, low income, having a language barrier, and lack of a case manager. Four of these were uniform with differences in the same direction across the three latent profiles. Low income was more likely to be reported by Black participants compared to White participants (79.3% vs 54.0% for those with LB, 80.5% vs 78% for those with HB-AV, and 82.3% vs 77.1% for those with HB-DAV). Lack of transportation was more common among Black participants (89.2% vs 68.6% among those with LB, 94.8% vs 93.7% among those with HB-AV, and 97.7% vs 91.1% among those with HB-DAV). White participants were more likely to report intimate partner violence (41.6% vs 37.0% among those with LB, 77.3% vs 63.5% among those with HB-AV, and 100% vs 76.0% among those with HB-DAV) and had higher discrimination scores (std mean: 0.38 vs 0.23 among those with LB, 1.69 vs 0.33 among those with HB-AV, and 7.79 vs 5.44 among those with HB-DAV) than Black participants. The remaining indicators that differed by race were nonuniform, meaning that they were more likely for some latent profiles but less likely for others. For example, food insecurity was higher among Black participants than White participants for the LB profile (std mean: 0.45 vs 0.17). However, this was reversed for the other profiles where Black participants had lower food insecurity than White participants (std mean: 1.24 vs 1.99 for those with HB-AV, 2.47vs 2.33 for those with HB-DAV). Another such pattern was observed for social support. For those with the LB profile, White participants had greater support compared to Black participants (std mean: 2.85 for White participants vs 2.48 for Black participants). This was reversed for the HB-AV (std mean: 2.22 for Black participants vs 1.74 for White participants) and HB-DAV (std mean: 2.22 for Black participants vs 1.57

for White participants) profiles. Other indicators with nonuniform profile differences by race were medical mistrust, access to care, unemployment, lack of a case manager, and language barriers.

Profile differences by southern/non-southern residence are shown in Figure 4. There were five indicators that showed uniform differences across all three latent profiles. Participants living in the Northern U.S. had higher drug use severity (std mean DAST score: 1.46 vs 1.23 among those with LB, 2.53 vs 2.16 among those with HB-AV, and 2.59 vs 2.14 among those with HB-DAV) and were more likely to have language barriers (21.4% vs 6.5% among those with LB, 20.5% vs 6.8% among those with HB-AV, and 18.2% vs 7.7% among those with HB-DAV) compared to participants living in the South. Southern participants had higher levels of medical mistrust (std mean: 3.76 vs 3.50 among those with LB, 3.87 vs 3.86 among those with HB-AV, and 4.43 vs 4.11 among those with HB-DAV) than participants living in the North. They were also more likely to be uninsured (48.4% vs 23.9% among those with LB, 29.9% vs 18.8% among those with HB-AV, and 37.4% vs 22.1% among those with HB-DAV) and lack case management services (84.1% vs 69.2% among those with LB, 67.1% vs 51.1% among those with HB-AV, and 62.4% vs 61.7% among those with HB-DAV) compared to those living in the North. Finally, discrimination differed by southern/non-southern residence but was nonuniform. It was higher among those living in the South among the LB (std mean: 0.29 vs 0.25) and HB-DAV (std mean: 5.90 vs 5.11) profiles. Conversely, discrimination was higher among those living in the North compared to those in the South among the HB-AV (std mean: 0.49 vs 0.39) profile.

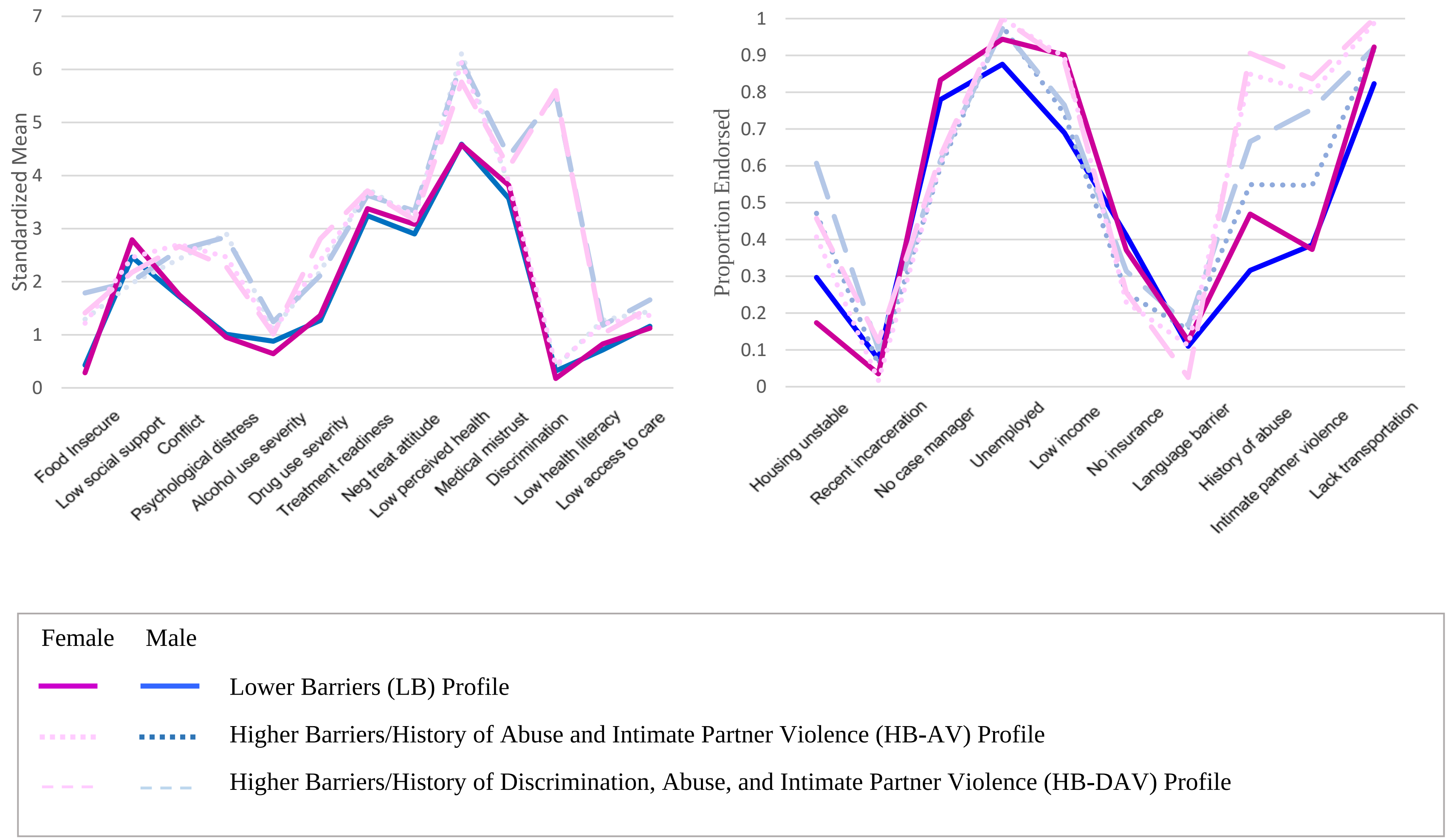

Figure S1. Differences in 3-Class Latent Profile Analysis model by gender.

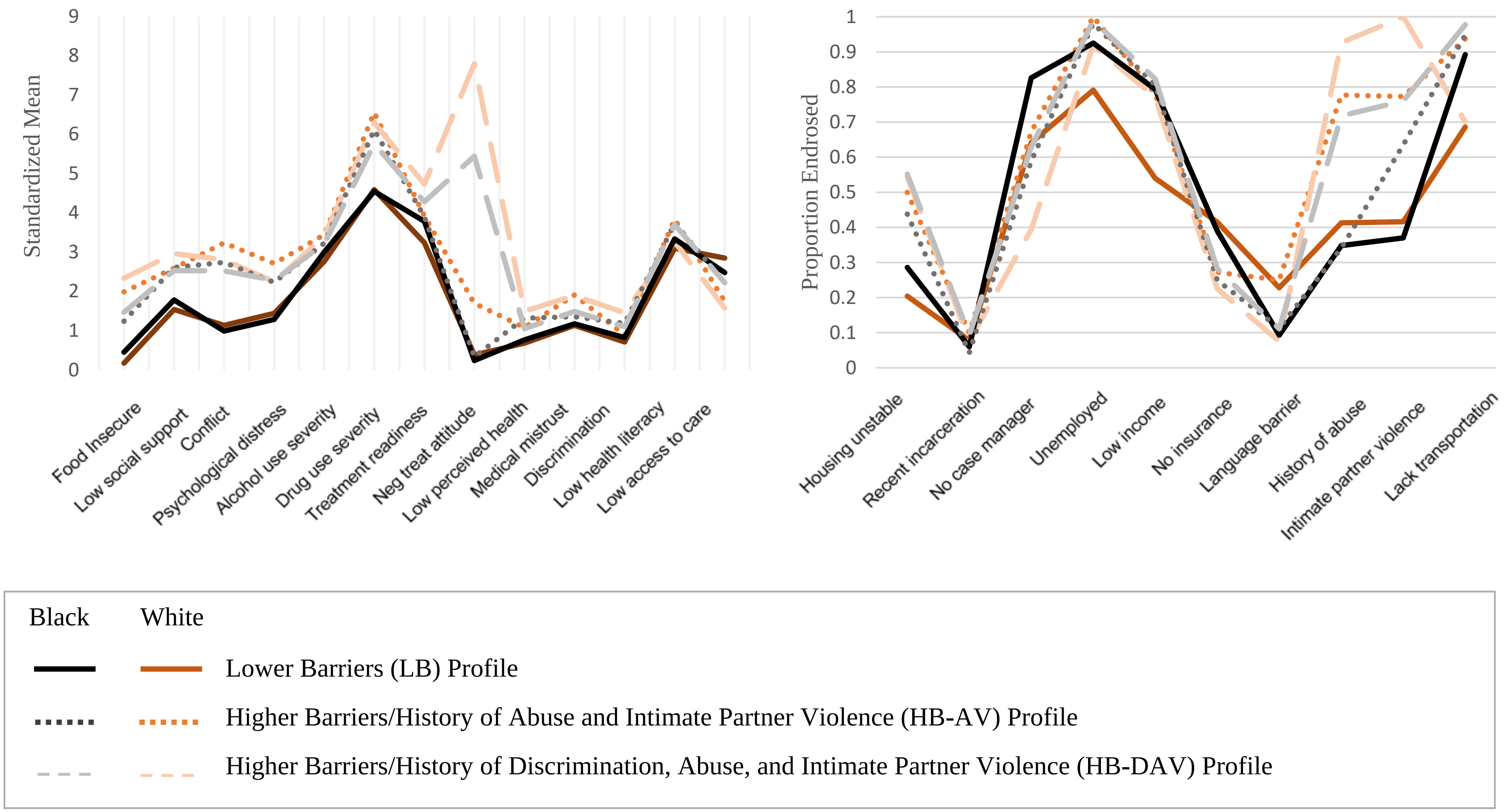

Figure S2. Differences in 3-Class Latent Profile Analysis model by race.

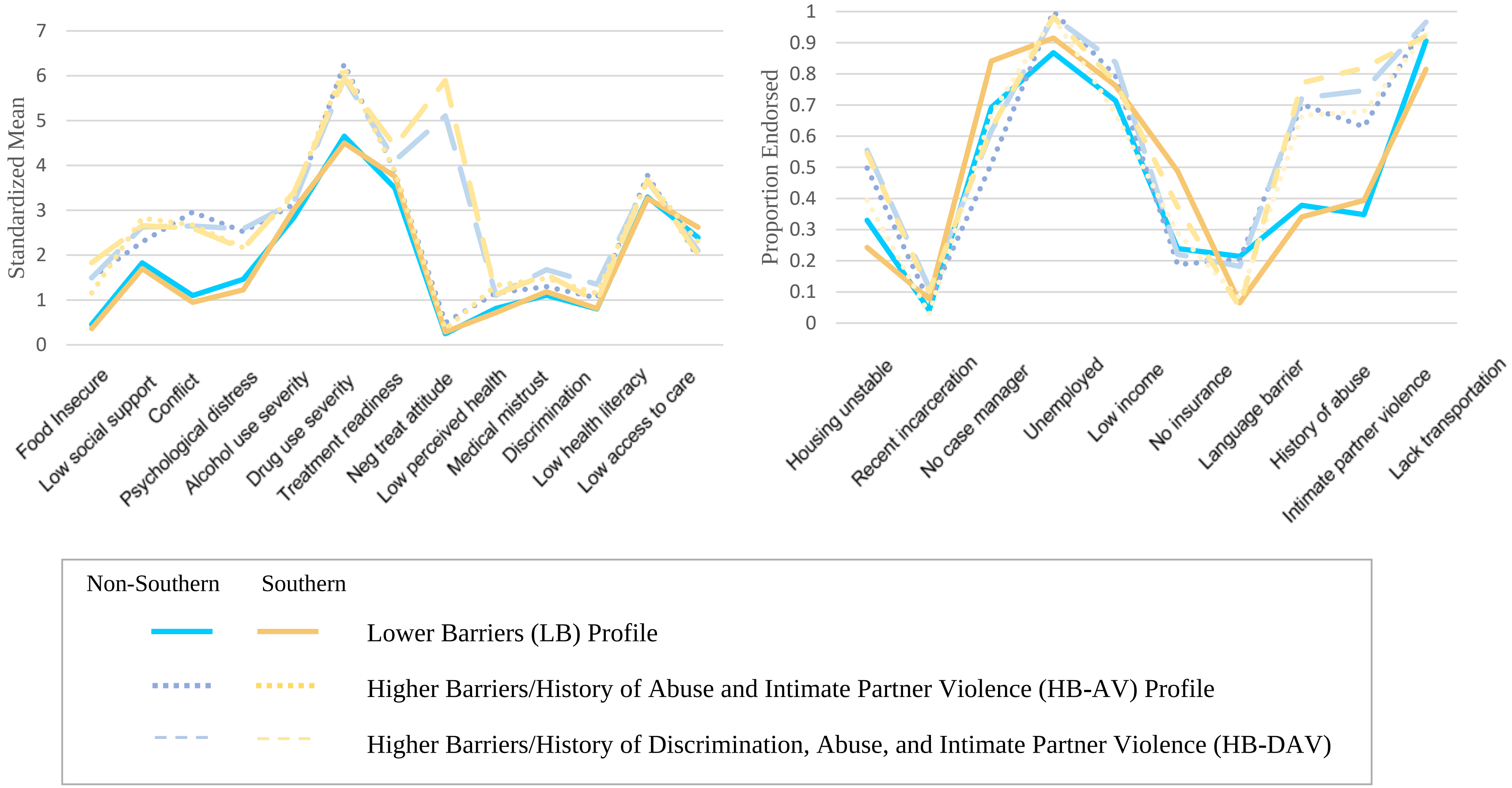

Standardized Mean
7
6
5
4
3
2
1
0
Food Insecure
Low social support
Conflict
Psychological distress
Alcohol use severity
Drug use severity
Treatment readiness
Neg treat attitude
Low perceived health
Medical mistrust
Discrimination
Low health literacy
Low access to care
Proportion Endorsed
1
0.9
0.8
0.7
0.6
0.5
0.4
0.3
0.2
0.1
0
Housing unstable
Recent incarceration
No case manager
Unemployed
Low income
No insurance
Language barrier
History of abuse
Intimate partner violence
Lack transportation
Non-Southern
Southern
Lower Barriers (LB) Profile
Higher Barriers/History of Abuse and Intimate Partner Violence (HB-AV) Profile
Higher Barriers/History of Discrimination, Abuse, and Intimate Partner Violence (HB-DAV)

Figure S3. Differences in 3-Class Latent Profile Analysis model by southern/non-southern residence.
